# Can Ultrasound and Contrast-Enhanced Ultrasound Help Differentiate between Subpleural Focal Organizing Pneumonia and Primary Lung Malignancy?

**DOI:** 10.3390/diagnostics12092074

**Published:** 2022-08-26

**Authors:** Ying Fu, Yutao Lei, Ligang Cui, Tingting Du, Fang Mei

**Affiliations:** 1 Department of Ultrasound, Peking University Third Hospital, Beijing 100191, China; 2 Department of General Surgery, Peking University Third Hospital, Beijing 100191, China; 3 Department of Pathology, Peking University Third Hospital, School of Basic Medical Sciences, Peking University Health Science Center, Beijing 100191, China

**Keywords:** contrast-enhanced ultrasound, organizing pneumonia, focal, primary lung malignancy, subpleural

## Abstract

Background: Subpleural focal organizing pneumonia (FOP) and primary lung malignancy (PLM) are usually confused. The aim of this study was to explore the value of ultrasound (US) and contrast-enhanced ultrasound (CEUS) in the differential diagnosis of FOP and PLM. Methods: A total of 23 patients (mean age: 64.57 ± 11.86 years) with FOP and 100 (mean age: 66.29 ± 11.05 years) with subpleural lesions diagnosed as PLM, confirmed by pathological diagnosis and clinical follow-up, were retrospectively enrolled. The largest lesion diameter, angle between the lesion border and thoracic wall, air bronchial sign, internal blood supply, blood supply form, and pleural effusion examined using conventional US were retrospectively analyzed. The indicators of CEUS included the arrival time of contrast agent in the lesion, lesion–lung arrival time difference, degree of enhancement, distribution uniformity of contrast medium, presence of non-enhancing region, and arterial filling mode in the lesion. A *p* < 0.05 was considered statistically significant. Results: Presence of air bronchial sign (odds ratio [OR] = 6.18, *p* = 0.025), acute angle between the lesion border and thoracic wall (OR = 7.124, *p* = 0.033), and homogeneous enhancement (OR = 35.26, *p* = 0.01) showed predictive value for the diagnosis of FOP. According to the results of the logistic regression analysis, the area under the receiver operating curve of the above features combined was 0.960, and the sensitivity and specificity were 95.0% and 82.6%, respectively. Conclusions: US combined with CEUS has the potential to differentiate between FOP and PLM.

## 1. Introduction

Organizing pneumonia (OP) is an uncommon pathological state characterized by Masson bodies filling the alveoli and spreading to the alveolar ducts and terminal bronchioles [1]. Masson bodies are formed by fibroblasts, myofibroblasts, and loose connective tissue [2,3,4]. According to etiology, OP can be divided into secondary OP caused by infection, connective tissue disease, injury, tumor, etc., or can be cryptogenic with unknown cause [5]. Approximately 10–15% of focal organizing pneumonia (FOP) cases present as a mass [6]. However, the lack of specific clinical symptoms and laboratory tests make it difficult to distinguish FOP from tumor diseases at an early stage. Therefore, it is important to analyze and summarize the imaging features of FOP to avoid unnecessary biopsy or surgical treatment [7].

High-resolution computed tomography (CT) is the recommended imaging technique for the diagnosis, assessment, and follow-up of OP [3]. CT presentation of OP is polymorphous, but a few patterns have recently been recognized as being more specific to this diagnosis, such as the burr sign, lobulation sign, bronchial inflation sign, and pleural indentation sign [8,9,10]. These signs are not unique to FOP, and there are a few overlapping signs between FOP and peripheral lung malignancy (PLM) on chest CT scans [11,12]. Furthermore, the role of positron emission tomography/CT in differentiating FOP from lung cancer may be limited [13,14].

In recent years, with the continuous development of ultrasound (US) technology and the increasing trend of lung cancer incidence rate annually, US and contrast-enhanced ultrasound (CEUS) have been widely reported in the study of subpleural space-occupying lesions and have achieved satisfactory results [15,16]. CEUS can dynamically observe the microblood supply to tissues in real time. This technique not only displays the blood supply distribution in the lesion, but also the dynamic vascular perfusion process of in-lesion and surrounding tissues in real time [17,18,19,20]. CEUS has achieved an accuracy rate of more than 90% in the differential diagnosis of benign and malignant lung lesions [15,16,21]. However, there are few studies on the US and CEUS features distinguishing FOP and PLM.

In this study, we retrospectively collected subpleural lesions of FOP and PLM to investigate the US and CEUS findings for the differential diagnosis of FOP.

## 2. Materials and Methods

### 2.1. Patients

**Figure 1:**
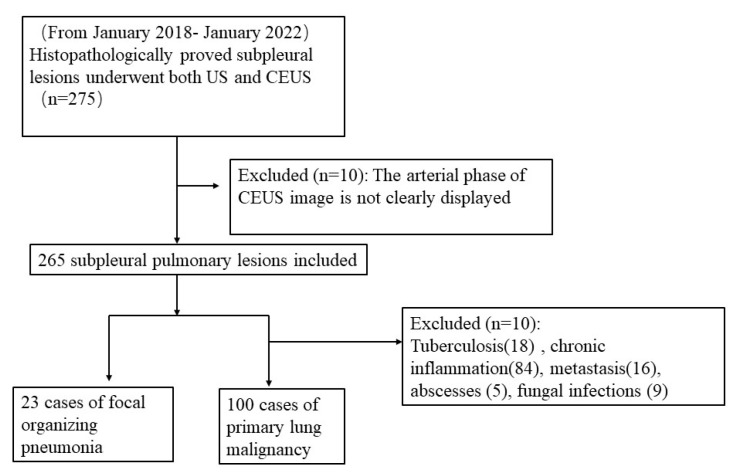
Flowchart of inclusion and exclusion criteria for focal organizing pneumonia and primary lung malignancy. (US: ultrasound; CEUS: contrast-enhanced ultrasound).

This study was approved by the institutional review board and ethics committee. A total of 275 pulmonary CEUS procedures were performed in our department between January 2018 and January 2022. Pathologic archives identified 23 patients with FOP and 100 with PLM concurrently (by either lobectomy or puncture biopsy) at our hospital. All patients had definite pathology results, and the CEUS video was clear and retrospective. The exclusion criteria were (1) incomplete clinical and ultrasonic data and (2) lung malignancy after treatment (Figure 1). In our study, a patient only selected one lesion for CEUS and puncture.

Among the patients with FOP, 12 had cryptogenic OP, 6 had secondary OP caused by autoimmune diseases, and 5 had secondary OP after infection. Among the patients with PLM, 68 had primary lung adenocarcinoma, 6 had small-cell lung cancer, 20 had lung squamous cell carcinoma, and 6 had sarcomatoid carcinoma. The clinical symptoms of the two groups were also summarized and compared.

### 2.2. Baseline US and CEUS

The GE Logiq E9 (GE Healthcare, Milwaukee, WI, USA) ultrasound instrument was used with a C5-1 probe for routine US and CEUS. A 3.0–5.0 mHz convex-array probe or a 2–9 mHz high-frequency linear-array probe was selected according to the size of the lesion. When suspected lesions were identified, the location, number, size, and ultrasonographic features of the nodules were evaluated. An appropriate scan section with the key target lesions was selected for CEUS. SonoVue (Bracco Co., Milan, Italy) was used as the contrast agent, and 2.0–2.4 mL of it was injected through the anterior elbow vein each time, followed by a 5.0 mL saline flush. The built-in timer of the ultrasound instrument was started when the injection of the contrast agent was initiated. The subpleural nodule area was continuously observed for 180 s. All static graphs and dynamic videos, except for the JPG or AVI format export, were saved for subsequent graph reading and data analysis.

The arrival time of contrast medium in the lesion, surrounding normal lung and chest wall tissue, the enhancement intensity and uniformity of contrast medium in the lesion, the presence or absence of non-perfusion area of contrast medium, and the entry mode of contrast medium in the lesion were observed frame by frame using film playback technology.

### 2.3. Data Analysis and Collection

The images and videos were reviewed by two ultrasonographers (working in consensus), each of whom had more than 10 years of experience with diagnostic US and was blinded to the patients’ clinical data, other imaging findings, and pathology results. If a controversial case existed, a senior ultrasonographer with 15 years of experience in CEUS would review the case and make a decision.

**Figure 2:**
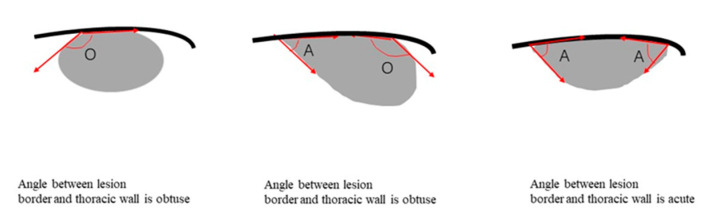
Illustration of the parameter of angle between lesion border and thoracic wall for subpleural pulmonary lesions. Black line represents the thoracic wall. Gray region represents the subpleural lesion. We drew an angle at the intersection of the edge of the lesion and the chest wall. As shown in the figure, “A” represents an acute angle, and “O” represents an obtuse angle. If one side of a lesion is an obtuse angle and the other side is an acute angle, the lesion is classified as obtuse.

Two-dimensional ultrasound and color Doppler images were used to record the size, presence of air bronchial signs, internal blood supply, blood supply form, and pleural effusion. An air bronchial sign was defined as punctate or linear hyperechogenicity in the consolidated lung. Blood supply forms were divided into single-phase wave arterial, three-phase wave arterial, venous, and mixed spectra. Based on other researchers’ investigations [22], we introduced the concept of the angle between the lesion border and thoracic wall. As long as one angle was obtuse, the lesion was classified as obtuse (Figure 2).

To reduce the error caused by the difference in individual circulation and injection speed of the contrast agent, a semi-quantitative method was adopted to define the supply artery [16]. An arrival time difference between the lesion and lung (lesion–lung difference) of ≤2.5 s was defined as the pulmonary artery supply; a lesion–lung difference of >2.5 s was defined as the bronchial artery supply. When the contrast agent in the lesion was enhanced later than that in the intercostal artery, regardless of whether the time difference was greater than 2.5 s, it was defined as the bronchial arterial supply.

**Figure 3:**
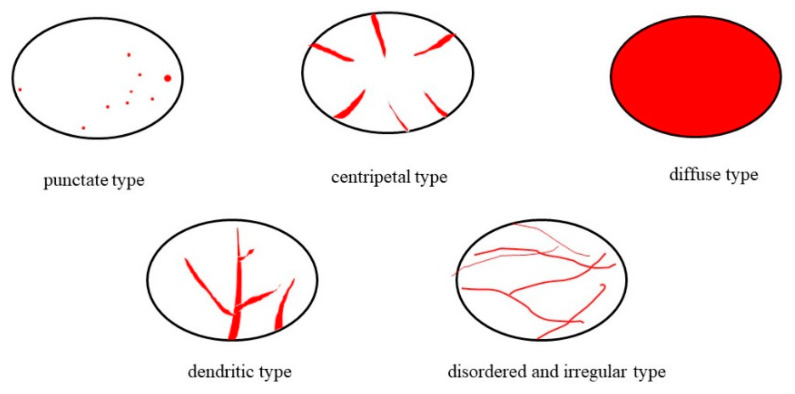
The entry mode of contrast medium at artery phase. The black circle represents the focus; red represents the distribution of contrast agent.

The entry mode of the contrast medium was divided into five types at the beginning of the enhancement: (1) punctate type: one or more local areas of the lesion showed punctate enhancement; (2) centripetal type: enhancement began at the periphery of the lesion and gradually extended to the center; (3) diffuse type: the entire lesion was uniformly enhanced; (4) dendritic type: contrast agent microbubbles began at the base of the lesion and developed into dendritic vessels, which enhanced and gradually expanded; and (5) disordered and irregular type: unrestricted vessel-like enhancement at multiple places [22,23] (Figure 3).

The uniformity of the distribution of the contrast medium in the lesion was classified as homogeneous or heterogeneous. Compared with peripheral lung tissue, enhancement intensity equal to or higher than that of peripheral lung tissue was defined as iso- or hyper-enhancement, respectively. An enhancement intensity lower than that of the peripheral lung tissue was defined as hypo-enhancement.

### 2.4. Reference Standards

Malignancy was confirmed via surgical resection or biopsy. A diagnosis of FOP was made via biopsy and follow-up for at least 6 months of imaging.

### 2.5. Statistical Analysis

The R language (version 4.0.3) was used for statistical analysis. Agreement between the 2 readers at CEUS was calculated and presented by Cohen’s kappa value (κ). A value of 0.81 or more represented excellent agreement. Quantitative data are presented as mean ± standard deviation and were compared using the independent samples *t*-test. If the data did not conform to a normal distribution, non-parametric analysis was used. Variance analysis was used to compare the count-data groups. We used a Chi-square test, continuity test, or Fisher’s exact probability test according to the data characteristics. Differences were considered statistically significant at *p* < 0.05. After univariate analysis, we performed multivariate analysis using binary logistic regression (forward stepwise method) on the variables showing significant differences. At the same time, we drew the receiver operating characteristic (ROC) curve and analyzed the diagnostic efficacy of each parameter and the best diagnostic threshold.

## 3. Results

### 3.1. Patient Characteristics

The FOP group comprised 12 men and 11 women (mean age, 64.57 ± 11.86 years) with 14 lesions on the right side and 10 lesions on the left side; the PLM group comprised 59 men and 41 women (mean age, 66.29 ± 11.05 years) with 43 lesions on the right side and 57 lesions on the left side. There were no significant differences in sex ratio or age composition between the two groups (*p* > 0.05). There was also no between-group difference in the distributions of symptoms between FOP and PLM patients (*p* = 0.276). See Table 1.

### 3.2. Conventional Ultrasonic Features

**Figure 4:**
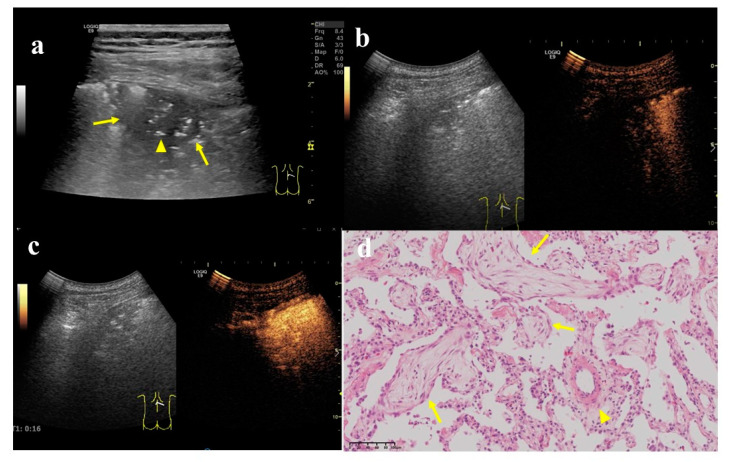
A 62-year-old female, who underwent total gastrectomy for gastric cancer 6 years ago and breast-conserving surgery for breast cancer 2 years ago, was coughing and expectorating for one month. (**a**) A wedge-shaped hypoechoic lesion (arrow) can be seen on the ultrasonic image of the right lung, with air bronchial sign inside (arrow head). Either angle between lesion border and thoracic wall was an acute angle. (**b**) After injection of contrast agent, the lesion began to enhance in 7 s, which was a uniform hyper-enhancement. The lung tissue began to enhance in 9 s, later than the lesion. (**c**) The enhancement of the lesion reached the peak in 16 s, and was a uniform enhancement. (**d**) An ultrasound-guided biopsy was performed. The pathological examination showed alveolar structure existed, the spacing was widened, and scattered lymphomonocytes could be seen in the focal alveolar cavity with cellulose exudation. Masson bodies (arrow) and small branch of pulmonary artery (arrow head) can be seen inside (×200). These findings conformed to the changes in organized pneumonia. After treatment with Medrol 8 mgqd for two months, the lesion became smaller and did not enlarge at the time of writing.

**Figure 5:**
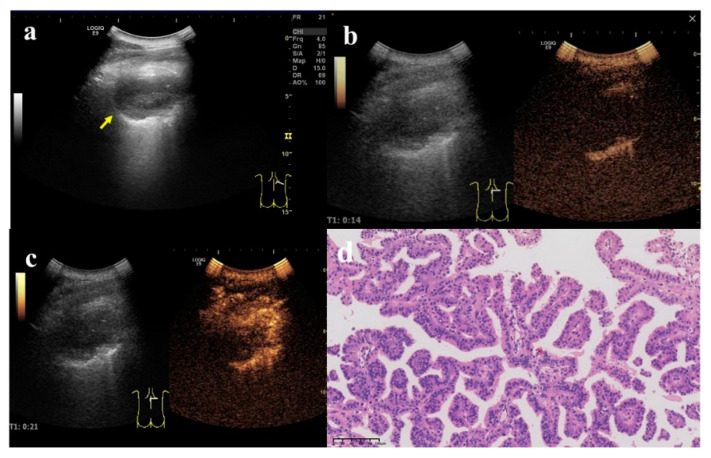
A 58-year-old male with a lesion occupying the deep space in the lower corner of the right shoulder armor. (**a**) Two-dimensional ultrasound showed a hypoechoic nodule, size 5.5 × 4.5 cm (arrow). At least one angle (between lesion border and thoracic wall) was obtuse. (**b**) Contrast-enhanced ultrasound showed that the enhancement time of lung tissue was 10 s and that of nodule was 14 s. (**c**) The enhancement began from the periphery to the center. The enhancement reached the peak in 30 s, and the enhancement degree was obviously uneven. (**d**) Pathologic evaluations revealed lung adenocarcinoma, mainly solid and acinar (×200).

Three out of six US parameters showed statistical differences between FOP and PLM (Table 2). The largest-diameter FOP lesion was smaller than that of PLM (3.92 ± 1.88 vs. 5.5 ± 2.91 cm, *p* = 0.002). Most of the angles between the lesion border and thoracic wall in FOP lesions were acute (12/23, 60.9%), whereas most of the malignant lesions were obtuse (68/100, 68%, *p* = 0.01). Air bronchial signs were more frequent in the FOP group than in the PLM group (*p* < 0.001). See Figure 4 and Figure 5.

There was no significant difference between the two groups for these features: pleural effusion, presence of rib invasion, presence of blood flow signal, and type of blood flow signal (*p* > 0.05).

### 3.3. CEUS Features

There were only five patients with disagreement between the two sonographers. The inter-reader agreement was excellent, with a κ equal to 0.854. There were statistical differences in lesion arrival time, major supply artery, degree of uniformity within the lesion, presence of perfusion area without contrast agent, and form of internal blood flow enhancement (Figure 4 and Figure 5). All FOPs were supplied by the pulmonary artery; that is, the lesion–lung difference was ≤2.5 s. Most PLMs were supplied by the bronchial arteries (55%, *p* < 0.001). Moreover, there were differences in the uniformity of the contrast medium distribution between the two groups (*p* < 0.001). Most of the FOP lesions were homogeneously enhanced (19/23, 82.6%). CEUS images in most patients in the FOP group showed diffuse enhancement (47.8%), while those in most patients in the PLM group showed centripetal enhancement (31%). The proportion of non-perfusion areas in the FOP group was significantly lower than that in the PLM group (2/23 vs. 40/100, *p* = 0.004; Table 3).

### 3.4. Multivariate Analysis

**Figure 6:**
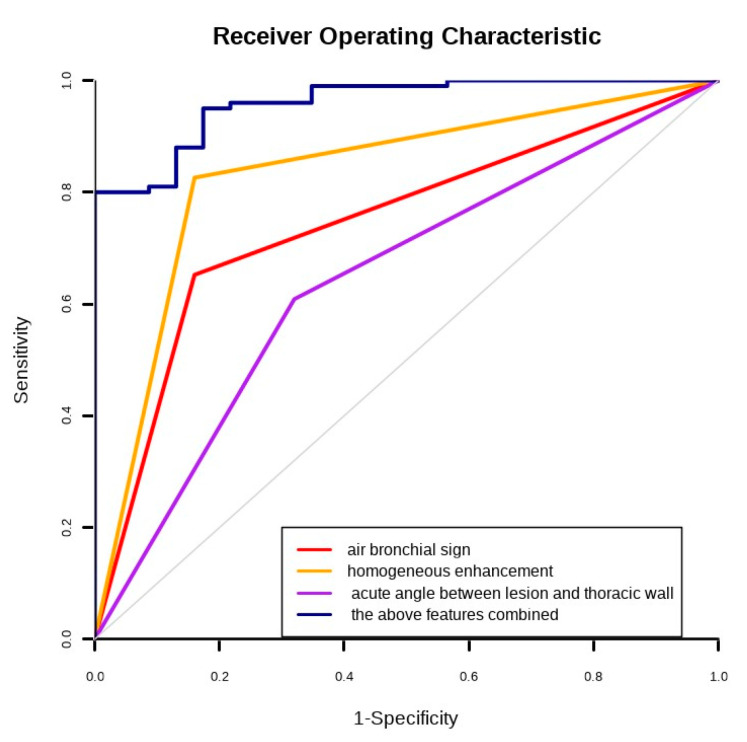
Receiver operating characteristic curves for air bronchial sign, homogeneous enhancement, acute angle between lesion border and thoracic wall, and the above features combined.

The parameters with *p* < 0.05 in univariate analysis were analyzed using logistic regression. The following US and CEUS indicators based on logistic regression showed significant predictive value for the diagnosis of FOP: presence of air bronchial signs (odds ratio [OR] = 6.18, *p* = 0.025), acute angle between the lesion border and thoracic wall (OR = 7.124, *p* = 0.033), and homogeneous enhancement (OR = 35.26, *p* = 0.01). According to the results of the logistic regression analysis, the area under the ROC of the above features combined was 0.960, and the sensitivity and specificity were 95.0% and 82.6%, respectively. The Youden index was 1.776 (Table 4 and Figure 6).

## 4. Discussion

FOP is a subtype of OP characterized by solitary and localized lesions. In previous studies, no specific features in clinical and radiological manifestations that distinguished FOP from other solitary pulmonary lesions, especially PLM, were found [6,24]. Traumatic surgical resection was the treatment method used. However, considering the benign nature of this disease, major pulmonary resection should be avoided [5]. Moreover, some reports have shown that standardized hormone therapy for FOP has a good prognosis [1,10].

Various diagnostic imaging methods have explored the characteristics of FOP and identified the distinguishing points between FOP and PLM. There are a few overlapping signs between FOP and PLM on chest CT scans, which makes it difficult to identify the two lesions in the lung using CT [10]. Some scholars have tried to use the energy spectrum to differentiate between PLM and FOP [11]. Although dual-energy spectral CT has the potential to identify bronchioloalveolar carcinoma and FOP, this technique is not generally used worldwide. High 18-fluorodeoxyglucose uptake in FOP patients may be related to inflammatory cell infiltration and local focal fibrosis [25], which limits its application.

Lung US examination is an effective alternative, especially for subpleural lesions, because it is a low-cost technique with no radiation, is highly portable, allows for repetition of examinations, and can be performed at the bedside [26]. CEUS increases the microvascular supply information of the lesion on the basis of two-dimensional US. The lung tissue was perfused with a dual blood supply. Due to the different origins of the blood vessels, the arrival time of the contrast agent from the pulmonary artery was earlier than that from the bronchial artery. This feature can be used to judge the blood supply artery of the lesion to further speculate on the nature of the lesion [16,27]. In addition to the difference in arrival time, semi-quantitative index time diagnostic criteria for malignant lesions (“lesion–lung arrival time difference ≥ 2.5 s”) have achieved high diagnostic accuracy (97.1%) for the differential diagnosis of subpleural benign and malignant lesions [16]. In our study, the arrival time and lesion–lung arrival time both showed statistical significance in univariate analysis. The arrival time of the FOP contrast agent was earlier, the time difference was <2.5 s, and the blood was supplied by the pulmonary artery, which is consistent with previous results. Multivariate analysis did not consider these features to be independent predictors of FOP and malignancy. The reason for this may be that the blood supply in lung malignancies is complex. Inflammation reactions in the tumor microenvironment contribute to tumor initiation, promotion, and progression [28]. Pulmonary-artery-derived blood supply may be involved in this process. When a malignant tumor develops, the blood supply from the bronchial arteries markedly increases and gradually replaces the supply from the pulmonary artery, becoming the main source of blood for the tumor. During the arterial phase of CEUS, pulmonary artery blood supply was detected in 45% of malignant lesions (45/100). This is one of the reasons why the ability of CEUS to distinguish community-acquired pneumonia from PLM in a large population has been questioned [28]. With the development of the disease course, the bronchial artery gradually participates in the replacement of the pulmonary artery. Meanwhile, the malignant cases in this group included some carcinomas in situ and small-cell lung cancer. The cancer cells spread and grow along the alveolar or bronchiolar wall, with less infiltration of the lung stroma and parenchyma, resulting in an enhancement phase similar to that in benign cases [29].

Except for arrival time and lesion–lung arrival time difference ≥ 2.5 s, the uniformity of contrast and the signs of two-dimensional ultrasound may have differential diagnostic ability. FOP was mainly homogeneous enhancement, accounting for 83.8%, while PLM was mainly heterogeneous enhancement, accounting for 55% (22/40, *p* = 0.003). This feature is one of the distinguishing points between FOP and PLM, confirmed by multivariate analysis, and the area under the ROC curve reached 83.3%. Generally, benign lesions are characterized by a dendritic distribution with little necrosis [23]. FOP is characterized by inflammatory cell infiltration, interstitial fibrous tissue, and fibroblast proliferation. Usually, there is a restricted-ventilation disorder without serious destruction of the lung tissue structure [24]. Therefore, the enhancement was homogeneous.

While malignant lesions are characterized by neovascularization, immature vessels are tortuous and irregular, and a large number of abnormal anastomotic and collateral branches are formed with uneven distribution [29]. The growth of tumor tissue is too fast and too large, which leads to insufficient blood supply to the tumor, and the tumor tissue will suffer from ischemic necrosis, showing heterogeneous enhancement [30]. Squamous cell carcinoma and sarcoma in malignant lung tumors are usually accompanied by necrosis, and CEUS shows non-perfusion areas in the lesion.

Nearly half of OP patients showed air bronchial signs on CT [28,31]. Ultrasonic air bronchial signs were found in 65.2% of the studies, which is similar to the results of the CT studies. The air bronchial sign indicates that the bronchioles in the focus are not destroyed or not completely destroyed, in turn indicating inflammatory obstruction. Masson’s corpuscles are formed in the alveoli and alveolar ducts, with or without terminal or respiratory intrabronchiolar polyps [1,24]. If there is a gas echo in the bronchioles that are not completely filled with polypoid tissue, an ultrasonic image can show the air bronchial sign. However, due to the long-term existence of chronic inflammation, the bronchial wall may be damaged, or the bronchiole cavity may be completely filled with inflammatory cells or liquid, resulting in the loss of the echo interface; therefore, two-dimensional ultrasound images do not show air bronchial signs. PLM shows infiltrative growth, invades and destroys the bronchial tube wall, and generally does not show air bronchial signs [8,9]. However, the mass compressed the tracheal cavity wall, which blocked the distal bronchus. The residual gas in the bronchus may cause air bronchial signs to appear, but its incidence is significantly lower than that of FOP.

Benign lesions are usually wedge-shaped, whereas malignant lesions are mostly spherical or irregular [22]. However, judgment of morphology is often subjective. Owing to the different imaging mechanisms, ultrasound cannot display the burr-pulling information of the nodule edge on the CT image because the boundary between the lesion and air-filled lung tissues is not clear in US images [32]. The angle between the lesion and the chest wall is easy to measure, and it is easier to summarize the difference between benign and malignant lesions using the two classification features. FOP lesions were mostly acute.

There are still some limitations to this study: (1) there are few cases included, and the diagnostic value of US and CEUS in FOP still needs to be confirmed by a large sample size; (2) it is only differentiated from PLM, and the key points of differential diagnosis with other special inflammatory lesions and neoplastic lesions need to be further studied and summarized; (3) this study only carried out the analysis of arrival time using a quantitative method and did not carry out the analysis of other parameters, which can be addressed in future research. Multicenter prospective studies using quantitative analysis should be carried out in the future.

## 5. Conclusions

In conclusion, the characteristics of ultrasound and CEUS for FOP were obvious. The presence of the air bronchial sign, acute angle between lesion border and thoracic wall, and homogeneous enhancement may provide a diagnostic basis for the differentiation between FOP and PLM.

## Figures and Tables

**Table 1:** Patient demographic and clinical characteristics.

Variable	FOP (*n* = 23)	PLM (*n* = 100)	Value	*p*
Sex (%)			0.357	0.55
Male	12 (52.2%)	59 (59.0%)		
Age (years)	64.57 ± 11.8614	66.29 ± 11.0543	−0.666 *	0.507
Symptoms (%)			1.1895	0.276
Asymptomatic	2 (8.7%)	18 (18.0%)		
Cough	15 (65.2%)	45 (45.0%)		
Fever	2 (8.7%)	8 (8.0%)		
Chest pain	4 (17.4%)	11 (11.0%)		
Weight loss	0 (0%)	18 (18.0%)		

FOP—focal organizing pneumonia; PLM—peripheral lung malignancy. * *t*-test value.

**Table 2:** Univariate analysis of ultrasound and doppler parameters.

Variable	FOP (*n* = 23)	PLM (*n* = 100)	Value	*p*
Lesion largest diameter	3.92 ± 1.88	5.5 ± 2.91	−3.231 *	0.002
Angle between lesion border and thoracic wall			6.657	0.01
Acute angle	14 (60.9%)	32 (32.0%)		
Obtuse angle	9 (39.1%)	68 (68.0%)		
Whether accompanied by pleural effusion			1.052	0.305
Yes	1 (4.3%)	15 (15.0%)		
No	22 (95.7%)	85 (85.0%)		
Air bronchial sign			24.028	<0.001
Present	15 (65.25)	16 (16.0%)		
Absent	8 (34.8%)	84 (84.0%)		
Whether blood flow is displayed			0.226	0.635
Yes	20 (87.0%)	80 (80.0%)		
No	3 (13.0%)	20 (20.05)		
Doppler spectra features			-	0.062
Single-phase wave arterial spectrum	7 (35.0%)	49 (61.3%)		
Three-phase wave arterial spectrum	11 (55.0%)	20 (25%)		
Venous spectrum	2 (10.0%)	7 (8.8%)		
Mixed spectrum	0 (0%)	4 (5.0%)		

FOP: focal organizing pneumonia; PLM: peripheral lung malignancy. * *t*-test value.

**Table 3:** Univariate Analysis of Contrast-Enhanced Ultrasound Parameters.

Variable	FOP (*n* = 23)	PLM (*n* = 100)	Value	*p*
Arrive time (s)	8.74 ± 4.22	11.81 ± 5.1	−2.525 *	0.017
Main blood supply artery			22.882	<0.001
Pulmonary artery	23 (100%)	45 (45.0%)		
Bronchial artery	0 (0%)	55 (55.0%)		
Degree of enhancement			0.098	0.754
Iso-/hyper-enhancement	22 (95.7%)	91 (91.0%)		
Hypo-enhancement	1 (4.3%)	9 (9.0%)		
Distribution of contrast agent			40.752	<0.001
Homogeneous	19 (82.6%)	16 (16.0%)		
Heterogeneous	4 (17.45)	84 (84.0%)		
Entry mode at artery phase			14.975	0.005
Punctate type	3 (13.0%)	6 (6.0%)		
Centripetal type	4 (17.4%)	31 (31.0%)		
Diffuse type	11 (47.8%)	19 (19.0%)		
Dendritic type	5 (21.7%)	18 (18.0%)		
Disordered and irregular type	0 (0%)	26 (26.0%)		
Non-perfusion area			8.149	0.004
Present	2 (8.7%)	40 (40.0%)		
Absent	21 (91.3%)	60 (60.0%)		

FOP: focal organizing pneumonia; PLM: peripheral lung malignancy. * *t*-test value.

**Table 4:** Independent Predictors of FOP From Combined use of US and CEUS Based on Logistic Regression.

Variables	Hazard Ratio (B)	Standard Error (Sx)	Wald Value	*p*	OR (95% CI)	Area	Sensitivity	Specificity	Youden Index
Homogeneous enhancement	3.433	1.118	9.438	0.01	35.26 [2.38, 522.65]	0.833	0.840	0.826	1.666
Air bronchial sign	1.759	0.825	4.546	0.025	6.9 [1.28, 37.18]	0.746	0.840	0.662	1.502
Acute angle	1.963	0.851	5.317	0.033	6.18 [1.16, 33.09]	0.644	0.680	0.609	1.289

FOP: focal organizing pneumonia; US: ultrasound; CEUS: contrast-enhanced ultrasound; OR: odds ratio; CI: confidence interval.

## Data Availability

The data that support the findings of this study are available on request from the corresponding author. The data are not publicly available due to privacy or ethical restrictions.
